# Clarity and adaptability of instructions preventing the spread of the COVID-19 virus and its association with individual and organisational factors regarding the psychosocial work environment: a cross-sectional study

**DOI:** 10.1186/s12913-023-10320-1

**Published:** 2023-11-28

**Authors:** Lena Marmstål Hammar, Moudud Alam, Caroline Eklund, Anne-Marie Boström, Annica Lövenmark

**Affiliations:** 1 School of Health, Care and Social Welfare, Mälardalen University, Västerås, Sweden; 2 Division of Nursing, Department of Neurobiology, Care Science and Society Karolinska Institute, Stockholm, Sweden; 3 School of Health and Welfare, Dalarna University, Falun, Sweden; 4 School of Information and Engineering/Statistics, Dalarna University, Falun, Sweden; 5 Theme Inflammation and Aging, Karolinska University Hospital, Huddinge, Sweden; 6 R&D unit, Stockholms Sjukhem, Stockholm, Sweden

**Keywords:** Nursing assistant, Care aide geriatric nursing, Work conditions, Occupational health, Residential facilities, Home care service, COVID-19

## Abstract

**Background:**

In Sweden, older people in residential care had the highest mortality rates, followed by those who received home care, during the coronavirus disease 2019 (COVID-19) pandemic. Staff working in the care of older people assumed responsibility for preventing the spread of the virus despite lacking the prerequisites and training. This study aimed to investigate the psychosocial work environment during the COVID-19 pandemic among staff in the care of older people and examine the factors associated with staff’s perceptions of the clarity of instructions and the ability to follow them.

**Methods:**

A cross-sectional study design was employed using a web survey. The staff’s perceptions of their psychosocial environment were analysed using descriptive statistics. The association between organisational and individual factors, as well as the degree of clarity of the instructions and the staff’s ability to follow them, were assessed using multivariate (ordinal) regression analysis.

**Results:**

The main findings show that perceptions of the clarity and adaptability of the instructions were primarily correlated with organisational factors, as higher responses (positive) for the subscales focusing on role clarity, support and encouragement in leadership at work were associated with the belief that the instructions were clear. Similarly, those indicating high job demands and high individual learning demands were less likely to report that the instructions were clear. Regarding adaptability, high scores for demands on learning and psychological demands were correlated with lower adaptability, while high scores for role clarity, encouraging leadership and social support, were associated with higher adaptability.

**Conclusions:**

High job demands and individual learning demands were demonstrated to decrease the staff’s understanding and adoption of instructions. These findings are significant on an organisational level since the work environment must be prepared for potential future pandemics to promote quality improvement and generally increase patient safety and staff health.

**Supplementary Information:**

The online version contains supplementary material available at 10.1186/s12913-023-10320-1.

## Introduction

Older persons were more severely affected during the coronavirus disease 2019 (COVID-19) pandemic, and in Sweden, older adults living in residential care facilities had the highest mortality rates, followed by those receiving home care services [1]. Staff working in the care of older people assumed responsibility for preventing the spread of the virus. Notably, residential care facilities and home care services in Sweden have been developed to manage such occurrences via strict hygiene or terminal care. However, most of the protective equipment were initially designated for hospital care, rendering residential care facilities and home care services poorly prepared to prevent the virus from spreading [2]. In addition, staff in the care of older people are mainly assistant nurses (AN) with basic care education at the upper secondary level or care aides (CA) without adequate formal education [3]. Their main assignments involve assisting patients in everyday living, such as through personal care, housekeeping, cooking and laundry. Home health care includes medical measures provided by health care professionals and commonly registered nurses and is regulated by the Swedish Health Care Act [4].

Due to the increasingly complex care needs amongst older persons in residential care facilities and home care services, staff are required to have a high level of knowledge and skills [5]. Given that registered nurses in the care of older people generally constitute only 5.3% of staff [2], they are unable to manage these tasks and must delegate medical tasks to other staff. In particular, home care service staff commonly work alone and must rely on their skills even in relation to advanced tasks for which they are not educated [6]. In addition, since the duties of ANs and CAs in home care services and residential care facilities are regulated by the Social Services Act [7], they are not supposed to be involved in medical care because they are not trained or educated for these types of tasks. This was posited as a major critique related to patient safety and security by the Corona Commission of Sweden [2]. Research has demonstrated that during the COVID-19 pandemic, staff working in health care generally experienced high levels of stress and anxiety, as well as depression and post-traumatic stress syndrome (PTSD) [8–11]. In residential care facilities and home care services, the staff suffered from anxiety, depression and post-traumatic stress syndrome due to poor working conditions and a lack of skills and knowledge regarding their job related to COVID-19 [12]. They also perceived themselves as abandoned, undervalued, unprotected and anxious about contracting COVID-19 themselves. This was also confirmed by the report from the Corona Commission of Sweden [2]. In addition, the staff found it difficult to follow the guidelines in preventing the spread of the virus; the rapid changes in the guidelines caused problems with converting them and educating the staff [2, 13].

Even before the pandemic, the care of older people in Sweden have already undergone changes in terms of organisation and regulations; in fact, the staff described increased job strain, physical and psychological stresses and mental exhaustion [5, 14–17]. Compared to 2005, the staff in 2015 had less education, met with a larger number of care recipients, received less support from managers and had a diminished ability to be involved in planning their work [5]. In summary, an increased workload and the need for higher competencies have resulted in increased job strain, thereby impacting the staff and their organisational work environment [5, 18–20]. Assander et al. [21] focused on the staff in home care services and found high job strain to be associated with leadership, organisational culture and climate and control at work. Job strain is a prevalent concept within the discourse of occupational stress [22, 23] and work-related stress, particularly within the research literature of nursing care [24, 25]. As delineated by the World Health Organization (WHO) [26], work-related stress arises when pressures and demands diverge from an individual’s competencies and knowledge, thereby challenging their coping strategies. The severity of work-related stress often intensifies when employees perceive an absence of support from their supervisors and colleagues as well as limited control over their work responsibilities. Karasek et al. [23] elaborated on the notion that increased job demands, autonomous working without adequate support and a sense of an inability to address issues during work contribute to increased levels of job strain. Within the Demand Control Support model [27], the primary foundations of work-related stress are identified as psychological demands (Demands), representing the psychological stressors associated with workload; decision latitude (Control) regarding one’s control over job-related decisions; and support or perceptions of the support received from superiors and colleagues. Dallner et al. [28] as well as and Lindström et al. [29] suggested psychological and social factors as fundamental aspects related to workplace stress. They also underscored more detailed aspects such as role clarity, learning demands, decision-making autonomy and empowerment and considered the positive challenges encountered within the work environment. The theories of Karasek, Dallner and Lindström [23, 28, 29] are in line with previous research [30–32], which shows that a balanced organisational structure, empowerment, psychological work environment and effective management are essential to foster a supportive work environment. In addition, as previously mentioned, the work environment in the care of older people before the pandemic showed that job strain is associated with lack of support and leadership, lack of education, time constraints and diminished ability to be involved in planning their work [5, 18–20]. It may be assumed that during the pandemic, the instructions to prevent rapid contamination changed. Due to the already stressful work environment, the low capability to educate the staff and the more advanced care the staff were required to perform, the clarity and the abilities to adapt to the instructions could be questioned.

In Sweden, no research has investigated individual and organisational factors related to the psychosocial work environment during the COVID-19 pandemic nor its relations to the clarity and adoptability of the instructions gained. We hypothesised that psychosocial factors, such as role clarity, positive challenges at work, encouraging leadership, demands, control over work and support, were associated with the perception of clarity of the instructions and ability to follow the instructions among staff in the care of older people during the COVID-19 pandemic. Therefore, this study aimed to investigate the psychosocial work environment during the COVID-19 pandemic among staff in the care of older people and examine the factors associated with the staff’s perceptions of the clarity of instructions and the ability to follow them.

## Methods

A cross-sectional study design was employed using a web survey.

### Data collection

Data were collected through a web-based questionnaire developed specifically for this study (supplementary file 1, questionnare) created in a web-based survey tool, which complies with the General Data Protection Regulation (GDPR). A link to the questionnaire was distributed via email as part of the monthly newsletters (April to September 2021) by the Swedish Municipal Trade Union (Kommunalarbetarförbundet) to its members working in the care of older people in Sweden (n ≈ 100,000). The questionnaire started with information about the study, research ethics and a consent letter. No incentive was offered to the participants for completing the survey. The survey was accessible between April and September 2021.

### Setting and sample

The eligible participants were ANs and CAs working in home care services or residential care for older people (municipal and private) in Sweden during the COVID-19 pandemic in the middle of 2021. ANs and CAs who did not work in the everyday care of older people, such as administrative staff, were excluded.

### Instruments

The survey consisted of two standardised instruments and two questions formulated specifically for this study and demographic information.

#### General nordic questionnaire for psychological and social factors at work (QPS_Nordic_^34+^)

*QPS*_*Nordic*_^*34+*^ measures the psychosocial aspects of the work environment. It is a general self-assessment instrument focusing on employees’ perceptions of their psychological and social work life, combined with the organisational work relationships within a person’s work life [28]. The QPS_Nordic_^34+^ consists of 37 questions and is divided into 23 subscales. Based on previous research, eight subscales, namely, *quantitative demands, demands on learning, positive challenges at work, role clarity, control over decisions, control over working pace, support from employers* and *encouraging leadership* were suitable and, thus, were used in our study. Each subscale included two items or questions, and the responses to the questions were based on a five-point Likert scale, ranging from 1 (very seldom/never) to 5 (quite to very often/always) [28, 33].

#### Demand-control-support questionnaire (DCSQ)

The DCSQ is a shorter and modified version of Karasek’s Job Content Questionnaire [23] and is a self-assessed 16-item questionnaire divided into three subscales: *psychological demands, decision latitude* and *social support* [34].

The responses in the subscales were based on a four-point Likert scale, ranging from 4 (agree/completely correct/often) to 1 (completely disagree/never at all). The single item regarding general health had response options on a five-point Likert scale, ranging from 1 (very good) to 5 (very poor).

#### Instructions and ability to prevent the spread of COVID-19 virus

As there are no validated measures focusing on the work environment for staff in the care of older people during the pandemic, the research team developed two questions for this study. The first question is: *Do you think you received clear instructions from the management to be able to prevent the spread of the virus in your workplace during the COVID-19 pandemic?* This was accompanied by the following response options on a five-point Likert scale: 1 (No, not at all) to 5 (Yes, completely). The second question is: *Do you think you have the ability to adopt the instructions from the management to be able to prevent the spread of the virus in your workplace during the COVID-19 pandemic?* The response options were based on a four-point Likert scale ranging from 1 (No, not at all) to 4 (Yes, completely).

Finally, data on demographic information were collected using questions about sex, age, mother tongue, job title, employer, employment contract and type and which region the respondent worked. See Table 1.

**Table 1:** Descriptive statistics from the respondents’ profiles (N = 983)

Variable/categories (statistics)	Values	Observed (n)	Missing instances (n_miss_)
Sex		975	8
Female (%)	89%	872	
Male (%)	11%	103	
Age groups (%):			
>= 20 and < 40 yrs.	22%		
>= 40 and < 55 years.	44%		
>= 55 and < = 68 yrs.	34%	805	178
Mother tongue		971	12
Swedish (%)	90%	871	
Non-Swedish (%)	10%	100	
Job title		981	2
Nursing assistant (%)	89%	871	
Care aides (%)	10%	106	
Annan (%)	1%	4	
Region		946	37
Stockholm (%)	11%	111	
Västerbottens (%)	16%	148	
Västra Götalands (%)	24%	228	
Södermanlands (%)	1%	7	
Other 18 regions	48%	452	
Employment contract		981	2
Permanent	94%	925	
Temporary	2%	21	
Payroll	4%	35	
Employment type		972	11
Home care (%)	34%	330	
Residential care (%)	65%	635	
Both (%)	1%	7	
Employer		977	6
Municipality (%)	84%	823	
Private (%)	12%	112	
Non-profit organisation (%)	4%	42	

Note: The figures in the ‘Values’ column were calculated after omitting the missing value (case-wise). n_miss_ = N-n

### Analysis

The staff’s perception of their psychosocial environment was analysed using descriptive statistics. Multivariate (ordinal) regression analysis was used to assess the association between the organisational and individual factors and the degree of clarity of the instructions and the ability to follow them.

The reliability of the instruments was assessed using Cronbach’s alpha statistics. The respondents’ profiles were summarised using descriptive statistics (relative frequency for categorical variables and mean for numeric variables). The staff’s perceptions of their psychosocial environment were summarised with descriptive statistics (mean, standard deviation [SD] and relative frequency of the response alternative). The effects of a set of individual characteristics (age, sex, work title, county, employer and employment contract) and organisational factors (*QPS*_*Nordic*_^*34+*^ and DSCQ indicators) on the response (dependent) variables, namely, (i) clear instructions and (ii) the ability to follow these instructions, were analysed using a proportional odds (PO; cumulative logit) model.

The dependent variables (i and ii above) were recorded using 5- and 4-point Likert scales, respectively, with a higher response category implying a negative response (unclear; not adaptable) and a lower response category implying a positive response to the respective survey questions. Given that the response variables are ordinal, a separate PO model [35] was fitted to infer the effects of the independent variables on each of the dependent variables. A set of predetermined independent variables of interest was included in the model, and the same set of independent variables was used in the PO model for each dependent variable. For the *QPS*_*Nordic*_^*34+*^ and DSCQ subscales consisting of multiple items, the respective mean item score for each respondent was used as the subscale score (independent variable) in the PO models.

In the PO analysis, the missing data were imputed using a multiple imputation method [36]. Missing data regarding the individual characteristics (e.g. sex, region, mother tongue, etc.) were not included. However, missing individual age was imputed because this variable contained the highest incidence of missingness (see Table 1). Responses conveying an unwillingness to answer a survey question (‘I don’t want to answer’ responses) were treated as missing data and were also imputed. The presence of missing data in at least one of the original variables was encountered in 42% of the original data instances (rows). Since any analysis based on complete cases would lose a large portion of the data (42%), the imputation of the missing values using the multiple imputation method was executed. Table 1 indicates that the missing data incidences were exceptionally low in the individual background variables, except for age (containing 18% missing).

All data analyses were performed in the R statistical software [37]. PO models were fitted using the ‘polr’ function from the ‘MASS’ library in R [35]. Missing data were imputed using the multiple imputation (number of imputation = 5) method, which was implemented via the ‘mice’ library [38]. Pooled parameter estimates and respective inferential statistics, after multiple imputations, have been reported (see Results). Statistical significance was assessed at the 5% level.

The instruments are demonstrated in a supplementary file 2 (Tables s1, s2, s3).

## Results

### Description of the sample and participants

In total, 1,114 individuals registered to answer the survey questionnaire, and based on the inclusion criteria, 983 persons were included in the analysis. Descriptive statistics regarding the respondents’ profiles are presented in Table 1. Most of the respondents were female (89%), and the age distribution was skewed to the left (mean age 49 years, median 51 years, 1st and 3rd quartiles were 41 years and max 56 years, respectively). The vast majority of the respondents were native Swedish speakers (90%), ANs (89%), permanently employed (94%) and employed by municipalities (84%). Four respondents identified themselves as neither NAs nor CAs and were omitted in the further analysis.

### Perceptions of the psychosocial environment

A summary of the respondents’ assessments of their psychosocial environment, with respect to the *QPS*_*Nordic*_^*34+*^ and DCQS indicators, is presented in supplementary file 2. All the *QPS*_*Nordic*_^*34+*^ and DCQS indicators were found to be reliable (Cronbach’s alpha > 0.7), with the exception of ‘decision latitude’ (alpha = 0.60; Table s2 in supplementary file 2). Overall, the respondents reported a high prevalence of quantitative demand, positive challenges at work, role clarity and support from employers (mean item score > 3 using a 5-point Likert scale). In more than 50% of the cases, the respondents answered in response class 3 (sometimes) or higher (often to highly often) for the respective questions (items) under these subscales. There was also a high average prevalence of strong ‘psychological demand’, ‘decision latitude’ and ‘social support’ in the sense that all but one item for these indicators had a mean score greater than 2 in a 4-point Likert scale. Perceptions of social support were highly positive (the subscale mean was slightly below 3 on a 4-point Likert scale).

### Clarity in the instructions

The results from the final PO models are displayed in Table 2 (for the dependent variable clarity) and Table 2 (for the dependent variable adaptability). Although all the independent variables have been retained (regardless of their statistical significance) in the fitted model, only those significant at the 10% level are presented (see Supplementary file 2, for a complete list). Given that the region variable consists of 22 regions, and the effects of most of the 21-region-specific dummies were statistically insignificant, they are omitted in the results (Tables 2 and 3).

**Table 2:** Summary statistics of the final PO model for clarity as the dependent variable (response order: mostly clear < no, to some extent unclear < no, very unclear < no, far from being clear)

Effect	Estimate	Std. error	*P*-value	COR = exp(-Estimate)
Mother tongue (ref. Swedish)				
Other	–0.43	0.24	0.07	1.54
Quantitative demands	0.20	0.10	0.05	0.82
Demands on learning	0.27	0.09	< 0.01	0.76
Role clarity	–0.33	0.08	< 0.01	1.39
Support from employer	–0.23	0.10	0.02	1.26
Encouraging leadership	–0.20	0.10	0.04	1.22
Psychological demands	0.31	0.17	0.07	0.73
Social support	–0.55	0.14	< 0.01	1.73

Note: COR = Cumulative odds ratio. COR > 1 implies positive association, meaning that the higher value of the respective independent variable (or compared with the reference category) results in higher cumulative odds of answering towards the lower response category. COR = 1 implies no effect. All significant effects at the 10% level are displayed, except for the geographical region

**Table 3:** Summary statistics from the PO model for adaptability as the dependent variable (response order: agree fully < agree to a good extent < no, somewhat disagree < no, do not agree at all)

Effect	Estimate	Std. error	*P*-value	COR = exp(-Estimate)
Employment contract (ref. permanent)				
Temporary	0.63	0.46	0.18	0.54
Payroll	–0.78	0.44	0.07	2.19
Demands on learning	0.22	0.09	0.02	0.80
Role clarity	–0.29	0.08	< 0.01	1.34
Encouraging leadership	–0.23	0.10	0.02	1.26
Psychological demands	0.72	0.18	< 0.01	0.49
Social support	–0.60	0.14	< 0.01	1.81

Note: Only significant effects at the 10% level are displayed in the table. The effects associated with all the (contrasting) categories from a categorical independent variable are displayed if one of the categories was found significant, except for the region effects, which are omitted anyway

The results in Table 2 indicate that the coefficient associated with the *QPS*_*Nordic*_^*34+*^ subscales ‘role clarity’ to ‘encouraging leadership’ and the DCQS subscale for ‘social support’ were negative (even the associated COR > 1), implying that higher values in these independent variables (compared with the reference category, the category in consideration) were associated with an answer towards the lower response category (i.e. respondents perceived the instructions to be clear). These effects were also statistically significant at a 5% level of significance. The other independent variables (quantitative demands, demands on learning) appeared to be negatively associated with the clarity of instructions, indicating that higher values in these independent variables were associated with dissatisfaction with the clarity of the instructions. The results (Table 2) reveal that a one-unit higher score for ‘demand on learning’ was associated with 24% lower cumulative odds of perceiving the instructions as clear, and this effect was statistically significant. A one-unit higher score for role clarity was associated with (statistically significant) 39% higher cumulative odds of perceiving the instructions as clear. A one-unit higher score for ‘social support’ was associated with (statistically significant) 73% higher cumulative odds of perceiving the instructions as clear. There were 22 regions, so discovering a few to be significantly different from others may be a random phenomenon (due to a type I error in the statistical significance test), implying that regional differences may be ignorable.

### Adaptability to instructions

The results in Table 3 indicate that a higher score for ‘demands on learning’ and ‘psychological demand’ is associated with (statistically) significantly lower adaptability (COR decreased by 20 and 51%, respectively, for a unit increase in the respective subscale score). Conversely, higher scores for ‘role clarity’, ‘encouraging leadership’ and ‘social support’ were associated with (significantly) higher adaptability (COR increase by 34%, 26% and 81%, respectively, for a unit change in these sub-scales).

## Discussion

This study aimed to investigate the psychosocial work environment during the COVID-19 pandemic among the staff in the care of older people and examine the factors associated with the staff’s perceptions of the clarity of instructions and the ability to follow them.

The main findings indicate that perceptions of the clarity and adaptability of the instructions were significantly positively correlated with organisational factors, namely, role clarity, support and encouragement in leadership at work. Likewise, respondents indicating high job demands, psychological demands and individual demands on learning had lower odds of answering that the instructions were clear. Davidson and Szanton [39] found similar results, suggesting that due to a lack of pandemic training and levels of competence and skills, the instructions and guidelines were difficult to understand and adopt. The Corona Commission of Sweden [2] also suggested that the instructions to prevent the spread of the virus were commonly changed rapidly, which jeopardised the organisations’ ability to coordinate commensurate care. Organisations lacked sufficient time to convert the instructions and continuously instruct all staff. During the pandemic, the already stressful work environment became even more strained [40, 41]; thus, the low adaptability of the instructions being associated with high quantitative demands and demands on learning was not surprising. Gray et al. [12] found that the strained situation, lack of skills and poor working conditions were correlated with anxiety, depression and PTSD during the COVID-19 pandemic. The conditions for the staff in the care of older people in Sweden have deteriorated in recent decades [15, 16]. The care of older people lacks the organisational prerequisites, resources and staff with the knowledge and education needed to face a pandemic, as concluded by the Corona Commission of Sweden [2]. Nevertheless, they were the ones who had to be responsible for not spreading the virus to the most vulnerable portion of the population in Sweden [1]. Even worse, the home care services staff worked alone and were accustomed to relying on their skills and facing advanced demands during work [6, 20]. The staff in home care services and residential care facilities were not involved in medical care [2] but had sole responsibility for the safety and security of the older people during the pandemic when even spouses or other relatives could not care for or interact with their loved ones.

The results also suggest that perceptions of clear instructions were mainly associated with role clarity, support and encouragement in leadership and social support, as was the ability to adopt the instructions associated with high scores in role clarity, encouraging leadership and support from employers. This finding is not surprising, as it is well known that these components are vital for a positive work environment. Even before the pandemic impacted Sweden, previous research has demonstrated that empowerment and effective management and leadership are essential [31]. How much more critical would it then be regarding empowerment and effective management and leadership for the staff during the pandemic [13] when facing unprecedented demands and stress in their working conditions?

Regarding adaptability, high scores for demands on learning and psychological demands were correlated with lower adaptability, while high scores for role clarity, encouraging leadership and social support were associated with higher adaptability. Given that the staff working in the care of older people lacked the medical competence needed during the pandemic, the demands on learning and psychosocial factors were not surprisingly correlated with low adaptability. Registered nurses with the necessary medical competence, even before the pandemic, were too scarce to be able to lead and instruct the staff and work bedside with older persons, so the staff were commonly left alone [2]. This was confirmed by previous research, indicating that the staff felt lonely, overly burdened with responsibilities and disregarded and undervalued, as they lacked adequate support from registered nurses and their managers [40]. In turn, the front-line managers oversaw a large group of staff (an average of 50 persons), so supporting each of them is challenging [3]. Similarly, a registered nurse in the care of older people in Sweden may be responsible for 400 to 500 older persons [42], so supporting the staff in their work is also highly challenging. As previously mentioned and suggested in the report of the Swedish Corona commission of Sweden [2], the need for more advanced care, and thus, higher medical competence, exists regardless of the presence of a pandemic; however, this intensified during the pandemic. The deficiencies in the care of older people in Sweden regarding low staffing, lack of staff continuity, inadequate competence and the inability to coordinate health care are strongly corroborated by research and have been known for decades. The Health and Social Care Inspection [43] previously revealed that the lack of registered nurses results in staff with low educational levels who are compelled to perform advanced assessments of the care needs of older persons, which severely jeopardises patient safety. This study does not address why the staff in other regions, compared with the Stockholm region, found the instructions less clear and had lower odds of adapting to the instructions, as the pieces of evidence from the data were insufficient to draw any strong conclusions on this issue. However, this illustrates the essential value of clear instructions to attempt to handle the tasks at hand. It is also difficult to know whether the respondents who stated that they understood the instructions but were unable to adapt the instructions meant that their work conditions hindered them from being able to perform the tasks required of them.

### Limitations

There are several strengths and limitations of this study. One limitation is the decision to use only eight of the possible 23 subscales of the *QPS*_*Nordic*_^*34*^ instrument. This choice was made to minimise the length of the questionnaire to lessen the risk that the participants may terminate their participation because excessive time is required to finish the questionnaire. We assessed the eight included subscales as the most relevant for this study. Notably, the instruments allowed different subscales to be used separately. However, this may have presented confounding variables that were not measured. The questions that constitute the dependent variables were not tested for validity. However, given that there were no validated measures focusing on this, we chose to develop these questions based on our expertise and previous research of work environmental care for older people, both preceding and during the COVID-19 pandemic. Unfortunately, an inadvertent discrepancy in the number of response alternatives emerged between these two inquiries, attributable solely to a technical oversight during the transition from a Word document to the questionnaire software programme. This, of course, represents a limitation inherent to the present study. However, we do not believe this has affected the analysis and, thus, not the results of our study.

The participants were asked to answer the questionnaire only once, but as it was an open questionnaire and we did not control for IP addresses, it is possible that the same person answered it multiple times. We also used a convenience sample recruitment strategy, heavily relying on the trade union members, and the survey participation was entirely voluntary, resulting in a response rate of about 1%, which is very low. Therefore, the sample may not be representative of the underlying population. However, about 80% of persons working in the care of older people in Sweden are members of a trade union, rendering our sample valid. It is also known that recruitment in this population is difficult and may have been more complex during the pandemic with the increased workload. However, it is evident that our findings are consistent with previous research regarding the work environment in the care of older people and with the Corona Commission of Sweden report. In addition, from a statistical point of view, it is important that the sample is randomly selected and that the sample size is large enough for the analysis. We believe we meet the second criterion, although the randomisation may be questionable. The distribution of the respondents across the regions may not be representative of the underlying population in the sense that Stockholm is the largest region in Sweden (with respect to the population), but only 11% of the respondents were from Stockholm. The highest number of respondents was from Västra Götaland (24%), while the lowest quantity was from Södermanland (1%).

The selected subscales from the *QPS*_*Nordic*_^*34+*^ overlap to some extent with the constructs measured in the DCSQ (demands, control and support). The decision to use the subscales from the *QPS*_*Nordic*_^*34+*^ was based on the fact that they more specifically measure modifiable factors relevant to the context of this study, whereas the DCSQ measures them on a higher abstraction level. For example, using the subscales ‘quantitative demands’ and ‘demands on learning’ from the *QPS*_*Nordic*_^*34+*^, the type of demand was assessed more specifically to understand what kind of demands contributed to the psychosocial work environment.

Some of the *QPS*_*Nordic*_^*34+*^ subscales were found to be highly correlated with the other subscales in the instrument; however, the variance inflation factor (VIF) did not indicate any possible multicollinearity issue with the PO models (max. VIF < 3.5). It was also observed that some subscales did not show a very high Cronbach alpha (min. alpha = 0.6; see supplementary file 2). However, in a non-clinical study, an alpha value of 0.7 (with a few as low as 0.6) may be acceptable [44]. Given that this study did not aim at developing instruments, we did not attempt to improve the alpha values.

The results from the PO models may be sensitive to the omitted confounding variables. Furthermore, it was not possible to carry out a likelihood test to assess the proportion odds assumption because the frequency of response to some response categories was low. We carried out the Brunt test (using the poTest function from the ‘car’ library in R [45] for testing the PO assumption, and an overall test could not reject the PO assumption. Even though the poTest pointed out a possible violation of the PO assumption by a few variables, such significant results can simply be due to a type I error in the test. However, we did not carry out any further investigation into this issue.

## Conclusions and implications

We can conclude that the staff in the care of older people during the pandemic experienced challenges in relation to organisational prerequisites, such as having a clear role, having control and receiving support from superiors. These results were identified in previous research even before the pandemic and in our case to understand and adopt instructions to prevent the spread of the COVID-19 virus. High job demands and individual demands on learning have been found to decrease the understanding and adoption of instructions. These findings are important on an organisational level because, when working with quality improvement, the work environment must be prepared for potential future pandemics. It is also important to increase patient safety and staff health.

### Electronic supplementary material

Below is the link to the electronic supplementary material.

Supplementary Material 1

Supplementary Material 2

## Data Availability

The data are not publicly available due to restrictions in accordance with the Swedish Public Access to Information and Secrecy Act (2009:400). Requests for access to the dataset should be sent to the corresponding author and will be considered by the University’s data protection officer.

## List of Abbreviations

AN: Assistant nurses
CA: Care aide
DCSQ: Demand-Control-Support Questionnaire
QPS_Nordic_: General Nordic Questionnaire for Psychological and Social Factors at Work
