# Supplementary material for: Clarity and adaptability of instructions preventing the spread of the COVID-19 virus and its association with individual and organisational factors regarding the psychosocial work environment: a cross-sectional study
Source: BMC Health Serv Res. 2023 Nov 28;23:1312. doi: 10.1186/s12913-023-10320-1 (PMC10683104; doi:10.1186/s12913-023-10320-1)
Supplement: Supplementary file 1 — Supplementary Material 1 [file 12913_2023_10320_MOESM1_ESM.docx]

**Supplementary file 1, Questionnaire**

This is how you respond to the questionnaire:

The following pages contain questions and statements about your work and your workplace. The purpose of this questionnaire is to collect your experiences of working in residential care (e.g. Nursing homes) and/or in Home care service during the Covid-19 pandemic in Sweden. This to build a basis to develop the work and the work environment for you who work in the care of older people.

Take your time when you answer. You answer most of the questions by selecting the answer option that best matches your opinion.

Example:

| Do you have to hurry to keep up with your work? | | | | | |
| --- | --- | --- | --- | --- | --- |
| Very seldom or never | Rather seldom | Sometimes | Rather often | Very often or always | Don’t want to anwer |
| 1 | 2 | 3 | 4 | 5 | 6 |

**Personal data**

Date of brith:

**Sex**

Male:

Female:

**Native langugare**

Swedish:

Other:

If other than Swedish, please state which:

**Professional Title**

Care Assistant:

Assistant Nurse:

Other: if yes, which?:

**In which city / Municipality do you work?**

**Where do you work?**

Home Care Service:

Residential care/ Nursing home:

Both:

If you are working in both Home care service and in Nursing homes, please answer the questions focusing on where you work the most

**Which employer do you have?**

Municipal employer:

Private employer:

Non-profit organisation:

**Working conditions**

Permanent employment:

Temporary employment:

Hourly employment:

**Do you have employment with more than one employer?**

Yes, one additional employer

Yes, more than one additional employer:

No

| **Is your workload irregular so that the work pile up?** | | | | |
| --- | --- | --- | --- | --- |
| Very seldom or never | Rather seldom | Sometimes | Rather often | Very often or always |
| **Do you have too much to do?** | | | | |
| Very seldom or never | Rather seldom | Sometimes | Rather often | Very often or always |
| **Are your work tasks too difficult for you?** | | | | |
| Very seldom or never | Rather seldom | Sometimes | Rather often | Very often or always |
| **Do you perform work tasks for which you need more training?** | | | | |
| Very seldom or never | Rather seldom | Sometimes | Rather often | Very often or always |
| **Are your skills and knowledge useful in your work?** | | | | |
| Very seldom or never | Rather seldom | Sometimes | Rather often | Very often or always |
| **Is your work challenging in a positive way?** | | | | |
| Very seldom or never | Rather seldom | Sometimes | Rather often | Very often or always |
| **Are there clearly defined goals in your work?** | | | | |
| Very seldom or never | Rather seldom | Sometimes | Rather often | Very often or always |
| **Do you know exactly what is expected of you at work?** | | | | |
| Very seldom or never | Rather seldom | Sometimes | Rather often | Very often or always |
| **Can you influence the amount of work assigned to you?** | | | | |
| Very seldom or never | Rather seldom | Sometimes | Rather often | Very often or always |
| **Can you influence decisions that are important for your work?** | | | | |
| Very seldom or never | Rather seldom | Sometimes | Rather often | Very often or always |
| **If needed, can you get support and help with your work from your immediate superior?** | | | | |
| Very seldom or never | Rather seldom | Sometimes | Rather often | Very often or always |
| **Are your work achievements appreciated by your immediate superior?** | | | | |
| Very seldom or never | Rather seldom | Sometimes | Rather often | Very often or always |
| **Does your immediate superior encourage you to participate in important decisions?** | | | | |
| Very seldom or never | Rather seldom | Sometimes | Rather often | Very often or always |
| **Does your immediate superior help you develop your skills?** | | | | |
| Very seldom or never | Rather seldom | Sometimes | Rather often | Very often or always |

| **Does your job require you to work very fast?** | | | | | | |
| --- | --- | --- | --- | --- | --- | --- |
| Often | Sometimes | | | Seldom | | Never/almost never |
| **Does your job require you to work very hard?** | | | | | | |
| Often | Sometimes | | | Seldom | | Never/almost never |
| **Does your job require too great effort of you?** | | | | | | |
| Often | Sometimes | | | Seldom | | Never/almost never |
| **Do you have sufficient time for all your work tasks?** | | | | | | |
| Often | Sometimes | | | Seldom | | Never/almost never |
| **Do conflicting demands often occur in your work?** | | | | | | |
| Often | Sometimes | | | Seldom | | Never/almost never |
| **Does your job require creativity?** | | | | | | |
| Often | Sometimes | | | Seldom | | Never/almost never |
| **Do you have the opportunity to learn new things in your work?** | | | | | | |
| Often | Sometimes | | | Seldom | | Never/almost never |
| **Does your job require doing the same task over, and over again?** | | | | | | |
| Often | Sometimes | | | Seldom | | Never/almost never |
| **Do you have the possibility to decide for yourself *how* to carry out your work?** | | | | | | |
| Often | Sometimes | | | Seldom | | Never/almost never |
| **Do you have the possibility to decide for yourself *what* should be done in your work?** | | | | | | |
| Often | Sometimes | | | Seldom | | Never/almost never |
| **It is a calm and pleasant atmosphere at my place of work** | | | | | | |
| Strongly agree | | Mildly agree | Mildly disagree | | Strongly disagree | |
| **There is a good collegiality at my work** | | | | | | |
| Strongly agree | | Mildly agree | Mildly disagree | | Strongly disagree | |
| **My colleagues are there for me (supports me)** | | | | | | |
| Strongly agree | | Mildly agree | Mildly disagree | | Strongly disagree | |
| **People at my work understand that I can have a “bad day”** | | | | | | |
| Strongly agree | | Mildly agree | Mildly disagree | | Strongly disagree | |
| **I get on well with my superiors** | | | | | | |
| Strongly agree | | Mildly agree | Mildly disagree | | Strongly disagree | |
| **I get on well with my co-workers** | | | | | | |
| Strongly agree | | Mildly agree | Mildly disagree | | Strongly disagree | |

| **Do you think that you received clear instructions from the management to be able to prevent the spread of the virus in your work during the Covid-19 pandemic?** | | | | | | |
| --- | --- | --- | --- | --- | --- | --- |
| Very little or not at all | Rather little | Somewhat | | Rather much | | Very much |
| **Do you think that you have had the ability to adopt the instructions from the management to be able to prevent the spread of the virus in your work during the Covid-19 pandemic?** | | | | | | |
| Very little or not at all | Rather little | | Rather much | | Very much | |

| **How do you assess your own health status?** | | | | |
| --- | --- | --- | --- | --- |
| Very good | Pretty good | Not good och bad | Pretty bad | Very bad |
