# Supplementary material for: Clarity and adaptability of instructions preventing the spread of the COVID-19 virus and its association with individual and organisational factors regarding the psychosocial work environment: a cross-sectional study
Source: BMC Health Serv Res. 2023 Nov 28;23:1312. doi: 10.1186/s12913-023-10320-1 (PMC10683104; doi:10.1186/s12913-023-10320-1)
Supplement: Supplementary file 2 — Supplementary Material 2 [file 12913_2023_10320_MOESM2_ESM.docx]

**Supplementary file 2, containing the dependent and independent variables of the study (tables s1-s3).**

Supplementary table s1

| QPS Subscales (IV) | Item wording | Cronbach’s  Alpha | Mean | SD | Reduced scale.  responses (%) | | |
| --- | --- | --- | --- | --- | --- | --- | --- |
|  |  | (α**)** |  |  | **1&2** | **3** | **4&5** |
| Quantitative demands |  | **0.86** |  |  |  |  |  |
|  | Is your workload irregular so that the work piles up? |  | 3.34 | 1.12 | 19.01 | 36.69 | 44.30 |
|  | Do you have too much to do? |  | 3.61 | 1.05 | 13.50 | 31.49 | 55.01 |
| Demands on learning |  | **0.73** |  |  |  |  |  |
|  | Are your work tasks too difficult for you? |  | 1.72 | 0.87 | 83.11 | 13.82 | 3.07 |
|  | Do you perform work tasks for which you need more training? |  | 2.11 | 1.12 | 64.85 | 24.46 | 10.69 |
| Positive challenges at work |  | **0.59** |  |  |  |  |  |
|  | Are your skills and knowledge useful in your work? |  | 4.47 | 0.90 | 4.84 | 6.39 | 88.77 |
|  | Is your work challenging in a positive way? |  | 3.53 | 1.09 | 15.50 | 33.16 | 51.33 |
| Role clarity |  | **0.81** |  |  |  |  |  |
|  | Are there clearly defined goals in your work? |  | 3.89 | 1.13 | 13.40 | 17.28 | 69.32 |
|  | Do you know exactly what is expected of you at work? |  | 4.42 | 0.88 | 4.35 | 8.70 | 86.96 |
| Control over decisions |  | **0.72** |  |  |  |  |  |
|  | Can you influence the amount of work assigned to you? |  | 2.23 | 1.18 | 63.41 | 21.48 | 15.11 |
|  | Can you influence decisions that are important for your work? |  | 2.77 | 1.13 | 40.58 | 34.27 | 25.16 |
| Control over working pace |  | **0.82** |  |  |  |  |  |
|  | Can you set your own work pace? |  | 2.36 | 1.23 | 57.08 | 24.02 | 18.89 |
|  | Can you decide yourself when you are going to take a break? |  | 2.37 | 1.23 | 55.57 | 25.57 | 18.87 |
| Support from employer |  | **0.88** |  |  |  |  |  |
|  | If needed, can you get support and help with your work from your immediate superior? |  | 3.24 | 1.31 | 28.88 | 27.22 | 43.90 |
|  | Are your work achievements appreciated by your immediate superior? |  | 3.00 | 1.37 | 37.96 | 25.34 | 36.70 |
| Encouraging leadership |  | **0.93** |  |  | | | |
|  | Does your immediate superior encourage you to participate in important decisions? |  | 2.85 | 1.36 | 41.96 | 26.72 | 31.32 |
|  | Does your immediate superior help you develop your skills? |  | 2.67 | 1.29 | 47.85 | 26.65 | 25.50 |

Note: Chronbach’s alpha was computed using polychoric correlation matrices of the items within each subscale. Missing values were deleted listwise when calculating each summary statistic.

Supplementary table s2

| DCSQ subscales and single items (IV) | Item wording | Cronbach’s  Alpha  (α) | Mean | SD | Reduced scale.  responses (%) | | |
| --- | --- | --- | --- | --- | --- | --- | --- |
|  |  |  |  |  | **1&2** | **3** | **4** |
| Psychological demands | Does your job require you to work very fast?  Does your job require you to work very hard?  Does your job require too great effort of you?  Do you have sufficient time for all your work tasks?  Do conflicting demands often occur in your work? | **0.88** | 3.16  3.06  3.02  2.30  2.90 | 0.70  0.72  0.78  0.94  0.80 | 12.94  16.87  21.63  60.02  27.11 | 55.95  57.00  51.29  28.41  50.36 | 31.11  26.13  27.09  11.57  22.52 |
| Decision latitude | Does your job require creativity?  Do you have the opportunity to learn new things in your work?  Does your job require doing the same task over, and over again? | **0.60** | 3.66  2.93  1.49 | 0.54  0.79    0.69 | 2.67  25.56  91.90 | 28.50  50.82  6.56 | 68.83  23.61  1.54 |
|  | Do you have the possibility to decide for yourself *how* to carry out your work?  Do you have the possibility to decide for yourself *what* should be done in your work? |  | 2.62  2.24 | 0.86  0.87 | 40.31  63.51 | 45.80  28.76 | 13.89  7.73 |
| Social support | It is a calm and pleasant atmosphere at my place of work  There is a good collegiality at my work  My colleagues are there for me (supports me)  People at my work understand that I can have a “bad day”  I get on well with my superiors  I get on well with my co-workers | **0.90** | 2.49  2.86  3.13  3.00  3.07  3.34 | 0.88  0.81  0.71  0.8  0.84  0.69 | 46.49  27.38  13.64  21.76  18.96  8.75 | 42.67  52.31  56.92  51.36  48.75  46.65 | 10.85  20.31  29.44  26.88  32.29  44.59 |
|  |  |  |  |  |  |  |  |

Supplementary table s3

| Single items  concerning Covid-19 (DV)  and  Health status (IV) |  | Item wording | Cronbach’s  Alpha  (α) | Mean | SD | Reduced scale.  responses (%) | | |
| --- | --- | --- | --- | --- | --- | --- | --- | --- |
|  |  |  |  |  |  | **1&2** | **3** | **4&5** |
| Concerning Covid 19  Health status |  | Do you think that you received clear instructions from the management to be able to prevent the spread of the virus in your work during the Covid-19 pandemic? |  | 2.05 | 1.14 | 77.26 | 9.16 | 13.58 |
|  |  | Do you think that you have had the ability to adopt the instructions from the management to be able to prevent the spread of the virus in your work during the Covid-19 pandemic?  How do you assess your own health status? |  | 1.83  3.57 | 0.80  0.97 | 83.44  16.78 | 12.39  21.19 | 4.17  62.13* |

* Single item on health status was answered in 5-point Likert scale (5 = Very good, … 1 = Very bad), and the response (%) in the last column (62.13) is combined relative frequency of response options 4 and 5.
